# Microdamage accumulation in the femoral head and its association with subchondral insufficiency fractures: A retrospective study

**DOI:** 10.1016/j.bonr.2026.101913

**Published:** 2026-03-26

**Authors:** Takahiro Negayama, Ken Iwata, Masashi Shimamura, Teppei Senda, Ryuichi Isozaki, Takanori Miki, Masakazu Ishikawa

**Affiliations:** a Department of Orthopaedic Surgery, Faculty of Medicine, Kagawa University, Kagawa, Japan; b Department of Orthopaedic Surgery, Shikoku Medical Center for Children and Adults, Kagawa, Japan; c Department of Anatomy and Neurobiology, Faculty of Medicine, Kagawa University, Kagawa, Japan

**Keywords:** Subchondral insufficiency fracture, Microdamage, Femoral head, Spinopelvic alignment, Pelvic tilt

## Abstract

**Introduction:**

Subchondral insufficiency fracture (SIF) of the femoral head typically occurs in older adults with compromised bone quality and often progresses to rapidly destructive coxarthropathy. However, the etiology of SIF remains poorly understood. We examined associations among spinopelvic alignment, pelvic tilt, and microdamage accumulation in the femoral head in patients with SIF compared with osteoarthritis (OA) secondary to hip dysplasia and cadaveric controls.

**Materials and methods:**

We analyzed eight SIF patients, ten OA patients, and nine cadaveric controls. All patients underwent total hip arthroplasty. Radiological evaluation included lumbar lordosis angle (LLA), sacral slope (SS), and pelvic tilt angles (PA) in standing and supine positions. Histomorphological assessment focused on a 5 × 5-mm cancellous bone area beneath the joint surface.

**Results:**

The SIF group exhibited significantly lower LLA and SS, as well as a greater difference between standing and supine PA compared with the OA group, indicating increased posterior pelvic tilt. Histomorphologic analysis revealed reduced trabecular bone volume and greater crack length in SIF compared with OA. Crack surface density was significantly higher in both SIF and OA groups than in controls.

**Conclusion:**

SIF patients demonstrated greater posterior pelvic tilt and lower LLA compared with OA. Histologically, SIF showed lower trabecular bone volume and longer microcracks. These data suggest the nature of the microdamage, not just the amount of the microdamage, is important to skeletal fragility. Our findings underscore the importance of assessing both spinopelvic alignment and bone quality when evaluating patients at risk for SIF.

## Highlights

• SIF patients showed greater posterior pelvic tilt and reduced lumbar lordosis
• SIF exhibited longer microcracks despite similar crack density compared to OA
• Crack length may be a better indicator of fragility than trabecular volume
• Spinopelvic alignment assessment is important for evaluating SIF risk
• Microdamage nature, not just amount, may contribute to skeletal fragility

## 1 Introduction

Subchondral insufficiency fracture (SIF) of the femoral head is a fragility-related fracture first described in 1996 (Bangil et al., 1996) and later characterized pathologically by Yamamoto et al. in 1999 (Yamamoto and Bullough, 1999). SIF typically affects older adults or individuals with compromised bone quality and frequently progresses to rapidly destructive coxarthropathy, ultimately requiring surgical intervention. (Onishi et al., 2022; Sonohata et al., 2021; Yamamoto, 2012; Chen et al., 2022) Although numerous clinical reports have examined SIF, studies integrating both spinopelvic alignment and histomorphometric evaluation remain limited, and the underlying mechanism of SIF is still not fully understood.

Posterior pelvic tilt has been reported to increase the loading stress on the femoral head and may contribute to the development of SIF (Kubo et al., 2020). Many studies have documented changes in pelvic tilt between supine and standing position. Whiles the typical change in Pelvic tilt angle (PA) is <10°; approximately 20% of individuals exhibit changes >10°, with some exceeding 30° (Uemura et al., 2017). Among pre-total hip arthroplasty (THA) patients, a posterior pelvic tilt >10° occurs in roughly 25% of the cases and is associated with advanced age, lumbar kyphosis, and reduced sacral slope (SS) (Tamura et al., 2014). This posterior pelvic tilt, frequently observed in pre-THA patients, can lead to insufficient acetabular coverage and increased joint contact pressure, thereby promoting the development of SIF (Jo et al., 2016; Ishihara et al., 2010).

Age-related osteoporosis is the most common cause of SIF (Chen et al., 2022; Kubo et al., 2018), but cases have also been reported in individuals with alkaptonuria (Hamada et al., 2014), renal and liver organ transplantation (Ikemura et al., 2005; Iwasaki et al., 2009; Higuchi et al., 2022), and tumor-induced osteomalacia (Chouhan et al., 2010), all of which involve impaired bone quality. In addition to reduced bone strength, elevated mechanical stress contributes to SIF pathogenesis. In particular, inversion of the labrum between the femoral head and acetabulum has been showed to increase focal stress concentration in affected patients (Fukui et al., 2014).

Histologically, femoral head in SIF typically show fractured granulation tissue, necrotic bone fragments, and disrupted trabeculae (Yamamoto and Bullough, 1999; Yamamoto et al., 2000). A case of SIF in osteogenic imperfecta further highlighted severe bone loss and accumulation of microdamage (Iwata et al., 2019). Despite these findings, the detailed pathophysiology of SIF remains unclear. Basic research exploring the etiology of SIF, especially studies combining histomorphometric analysis and spinopelvic alignment assessments, are scarce.

The aim of this study was to investigate the spinopelvic alignment, pelvic tilt, and the accumulation of microdamage in the femoral head in patients with SIF, and to compare these findings with those from patients with osteoarthritis (OA) caused by developmental dysplasia of the hip (DDH) and with cadaveric controls, to better elucidate the etiology of SIF. We hypothesized that SIF patients would exhibit greater posterior pelvic tilt and increased microdamage accumulation, with distinct crack morphology, compared with OA patients and cadaveric controls.

## 2 Methods

### 2.1 Ethics statement

The study protocol was approved by the Institutional Review Board of Kagawa University (approval code: 29–213). Written informed consent was obtained from all participants prior to enrollment. For cadaveric specimens, appropriate consent for tissue donation and research use was obtained in accordance with institutional and national regulations.

### 2.2 Patients

This retrospective observational study employed three groups: patients with SIF of the femoral head, patients with OA secondary to DDH, and cadaveric controls without OA.

Between 2006 and 2013, eight hip joints in eight patients diagnosed with SIF underwent total hip arthroplasty (THA). The diagnosis of SIF was based on established criteria from previous reports (Yamamoto, 2012; Chen et al., 2022) and confirmed by magnetic resonance imaging (MRI) findings. Consecutive sampling was used, and all patients who met the following criteria during the specified period were included: (1) MRI-confirmed diagnosis of SIF, (2) minimum follow-up of 2 years, and (3) treatment with THA. This approach was chosen to minimize selection bias within our institution's patient population. Patients who had received teriparatide treatment were excluded because of its potential influence on bone metabolism. Cases with incomplete clinical information – defined as missing preoperative imaging or incomplete follow-up records – were also excluded to ensure analytical integrity.

**Fig. 1:**
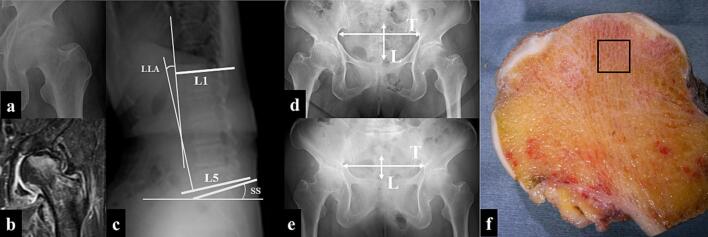
A 74-year-old woman presented with a subchondral insufficiency fracture of the left femoral head. (a) Radiograph showing femoral head osteopenia. (b) T2-weighted fat-suppressed magnetic resonance imaging showing a low-intensity band pattern and femoral head collapse. (c) Standing lateral spine X-ray illustrating the lumbar lordosis angle (LLA) and sacral slope (SS) measurements. (d) Supine pelvic anteroposterior view showing the pelvic diameter measurements (T and L) and femoral head collapse. (e) Standing pelvic radiograph demonstrating posterior pelvic tilt. (f) Coronal slice of the femoral head indicating the region assessed for tissue morphometry and microdamage. The femoral head exhibited defects in the cartilaginous layer at the weight-bearing surface and exposed subchondral bone.

Diagnostic criteria of SIF included the absence of evident radiographic abnormalities at symptom onset of pain aside from osteopenia (Fig. 1a) and subchondral, low-intensity band parallel to the articular surface on T2-weighted fat-suppressed MRI within an area of the bone marrow edema (Fig. 1b).

For comparison, propensity score matching identified 10 age- and sex-matched hips from 10 patients who underwent THA for OA secondary to DDH without prior hip surgery; these patients comprised the OA group. None of the patients had received preoperative osteoporosis treatment, and all participants were women. Nine femoral heads from cadavers without OA served as controls. Cadaveric donors were selected based on age to match the patient groups. Detailed clinical background information, including medical history, was unavailable for the cadaveric donors.

### 2.3 Radiological evaluation

Preoperative radiological assessments included evaluation of the lumbar lordosis angle (LLA), sacral slope (SS), and pelvic tilt angles (PA) in both standing and supine positions. LLA was defined as the angle between the superior margin of the first lumbar vertebra and the inferior margin of the fifth lumbar vertebra on a standing lateral radiograph of the spine. SS was measured as the angle between the upper edge of the sacral vertebrae and the horizontal plane on a standing lateral spinal radiograph (Fig. 1c). PA was calculated based on the method previously described (Taki et al., 2012), using standing and supine pelvic radiographs. The transverse diameter of the pelvis (T) was defined as the greatest transverse width of the pelvic ring, while the longitudinal diameter (L) was defined as the vertical distance between the line connecting the lower ends of the right and left sacroiliac joints and the line connecting the upper margin of the pubic symphysis (Fig. 1d, e). For women, the formulae used to calculate PA were as follows:

PA=−69×L/T+61.6.

Cadaveric donors were included only in the histomorphometric analyses and not in the radiological assessments, as preoperative imaging was unavailable for this group.

### 2.4 Histological evaluation of bone microdamage

The femoral heads obtained during surgery were sectioned coronally to a width of 15 mm at the center. The specimens were then stained *en bloc* with 1% basic fuchsin and embedded in methyl methacrylate following the protocol established by Burr and Stafford (Burr and Stafford, 1990). To assess the microdamage and histomorphology, 100-μm-thick ground sections were prepared. Tissue morphometry and microdamage were evaluated within a 5 × 5-mm region of cancellous bone located at least 3 mm beneath the loading joint surface (Fig. 1f). The upper yellow square, as defined by Fazzalari et al., corresponds to the subchondral primary compression zone (Fazzalari et al., 1989; Fazzalari et al., 2002).

Histomorphometric measurements were performed using a semi-automated digitizing image analyzer consisting of a light or epifluorescent microscope (NIKON SOLUTIONS CO., LTD., Tokyo, Japan) at 100 × magnification and a digitizing pad connected to a computer running the histomorphometric software (System Supply Co., Nagano, Japan). All the measurements were conducted by a single histomorphometrist in a blinded manner, and parameters were defined according to the American Society for Bone and Mineral Research Nomenclature Committee guidelines (Dempster et al., 2013).

Bone microdamage was identified by the presence of characteristic fracture-shaped features with a defined depth of field and a halo of heightened basic fuchsin staining. Quantitative microdamage parameters included mean crack density (Cr.Dn = Cr.N/B.Ar (#/mm²)), crack length (Cr.Le (μm)), and crack surface density (Cr.S.Dn = Cr.N × Cr.Le/B.Ar (μm/mm²)). Trabecular structure was assessed by measuring the trabecular bone volume (BV/TV, %). These methods followed established procedures described previously (Shimamura et al., 2019).

### 2.5 Statistical analysis

All statistical analyses were performed using EZR (Saitama Medical Center, Jichi Medical University, Saitama, Japan) (Kanda, 2013). Data are reported as the mean ± standard deviation. Radiological parameters were compared between groups using the Mann-Whitney *U* test. Microdamage and histomorphometric variables were compared using analysis of variance, followed by Tukey's multiple comparison test when significant differences were identified. Statistical significance was defined as *p* < 0.05.

## 3 Results

### 3.1 Patient demographics

There was no significant difference in age between the SIF and OA groups at the time of THA (*p* = 0.13) (Table 1).

**Table 1:** Demographics of the SIF, OA, and control groups.

	SIF (*n* = 8)	OA (*n* = 10)	CNT (*n* = 9)	*P*-value
Age (years)	74.0 ± 10.7	71.9 ± 9.5	83.1 ± 12.5	0.13
Sex (male/female)	0/8	0/10	0/9	1.0

Data are presented as the mean ± standard deviation or number.

### 3.2 Radiographic findings

The radiographic findings are summarized in Table 2. The LLA was significantly lower in the SIF group compared with the OA group (14.0 ± 17.3° vs. 35.6 ± 18.4°, *p* = 0.009). SS was also significantly reduced in the SIF group relative to the OA group (17.8 ± 17.7° vs. 31.1 ± 9.6°, *p* = 0.04). There was no significant difference in supine PA between the SIF and the OA groups (*p* = 0.47). In contrast, standing PA was significantly greater in the SIF than in the OA groups (47.7 ± 12.8° vs. 33.5 ± 10.6°, *p* = 0.044). Moreover, the changes in PA from supine to standing differed significantly between the two groups (*p* = 0.005), with an angle of 15.8 ± 7.6° in the SIF group compared with 5.4 ± 3.8° in the OA group.

**Table 2:** Radiographic findings in the SIF and OA groups.

	SIF (n = 8)	OA (n = 10)	P-value
LLA (°)	14.0 ± 17.3	35.6 ± 18.4	**0.009**
SS (°)	17.8 ± 17.7	31.1 ± 9.6	**0.043**
Supine PA (°)	31.9 ± 7.9	28.1 ± 8.2	0.470
Standing PA (°)	47.7 ± 12.8	33.5 ± 10.6	**0.044**
∆PA (°)	15.8 ± 7.6	5.4 ± 3.8	**0.005**

Data are presented as the mean ± standard deviation or number.

LLA, lumbar lordosis angle*;* SS, sacral slope; PA, pelvic tilt angle; ∆PA, difference between the standing and supine PA.

### 3.3 Histomorphology and microdamage findings

**Fig. 2:**
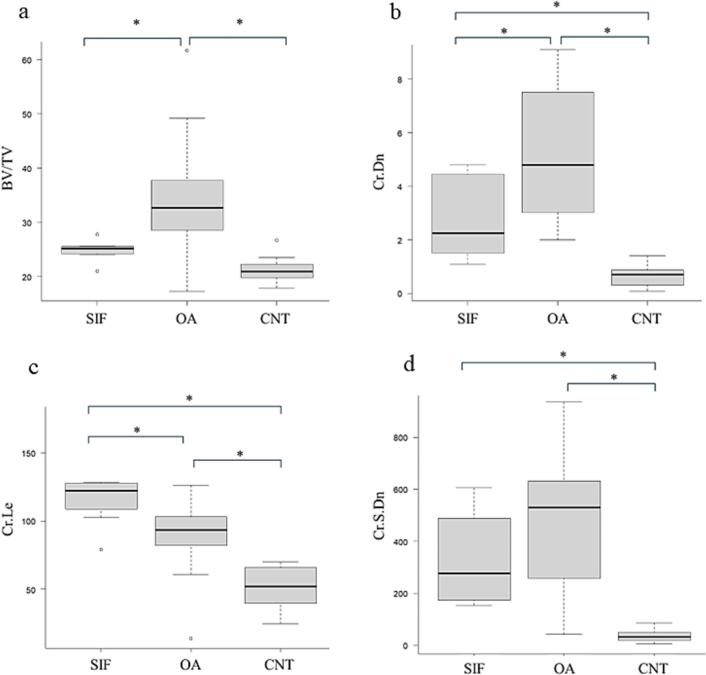
Histomorphometric analysis of the femoral head bone in the subchondral insufficiency fracture (SIF), osteoarthritis (OA), and control (CNT) groups. Box plots show (a) trabecular bone volume fraction (BV/TV, %), (b) crack density (Cr.Dn, #/mm^2^), (c) crack length (Cr.Le, μm), and (d) crack surface density (Cr.S.Dn, μm/mm^2^). Boxes represent the interquartile ranges with median lines. Whiskers extend to minimum/maximum values, excluding outliers (individual points). *Statistically significant difference (*p* < 0.05).

The histomorphology and microdamage results are summarized in Table 1 and Fig. 2. Trabecular bone volume (BV/TV) was 24.8 ± 1.78% in the SIF group, 35.0 ± 12.5% in the OA group, and 21.1 ± 2.8% in the control group, with the OA group demonstrating significantly higher BV/TV than the other two groups (*p* < 0.0001).

Crack Density (Cr.Dn) was 2.8 ± 1.5 in the SIF group, 5.3 ± 2.4 in the OA group, and 0.6 ± 0.4 in the control group, showing a significant difference among all the groups (*p* < 0.0001). Cr.Dn was significantly greater in the OA group compared with both the SIF and control groups.

Mean crack length (Cr.Le) was the highest in the SIF group (123.4 ± 29.9), followed by the OA group (84.2 ± 29.9) and controls (50.4 ± 15.8), with the SIF group demonstrating significantly longer crack than the other two groups (*p* < 0.0001).

Crack surface density (Cr.S.Dn), the product of crack number and length standardized to bone area, was 329.1 ± 168.3 in the SIF group, 475.2 ± 255.2 in the OA group, and 35.6 ± 25.1 in the control group. Both the SIF and OA groups showed significantly greater microdamage accumulation compared with the control group (p < 0.0001).

**Fig. 3:**
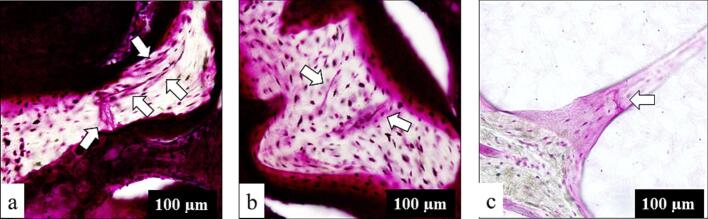
Photomicrographs of a femoral head stained with basic fuchsin to show the microdamage. (a) In subchondral insufficiency fracture, multiple microcracks (arrows) are connected within the thin trabeculae, forming a long crack. (b) In osteoarthritis, several short microcracks (arrows) are observed within the thick trabeculae. (c) In the control, a single microcrack (arrow) is visible within the thin trabecular bone. Scale bar, 100 μm.

Histological observations supported these quantitative findings. In the SIF group, multiple microcracks propagated through thin trabeculae, forming long, continuous cracks (Fig. 3a). In the OA group, several short microcracks were present within the thickened trabeculae (Fig. 3b). Control samples exhibited only isolated microcracks within thin trabecular bone (Fig. 3c), a pattern consistently observed across all nine control femoral heads, which demonstrated minimal microcrack formation in controls. These patterns suggest that fragile trabeculae in SIF allow microcracks to connect and propagate, resulting in longer cracks, whereas the thicker trabeculae in OA can accommodate multiple shorter microcracks without propagation.

## 4 Discussion

In this study, patients with SIF had increased posterior pelvic tilt and reduced lumbar lordosis compared with the OA group, consistent with previous reports. Histomorphological analysis further revealed that SIF was characterized by reduced trabecular bone volume and greater crack propagation compared with OA. To our knowledge, this study is the first study to demonstrate an association between spinopelvic alignment and increased microdamage in the femoral head, providing new insight into the pathogenesis of SIF.

The case of a 74-year-old woman with SIF exemplifies these findings. Radiographs showed an 11° difference between standing and supine PA, indicating increased posterior pelvic tilt in the standing position and an LLA was 6°, reflecting a decrease in lumbar lordosis. These spinal and pelvic alignment changes corresponded with progressive collapse of the weight-bearing region of the femoral head over several months. T2-weighted fat-suppressed MRI revealed fracture lines and bone marrow edema in the weight-bearing area. Gross examination of the resected femoral head showed cartilage defects and exposed subchondral bone on the weight-bearing surface, while histological analysis identified long microcracks within thin trabeculae.

In the present study, the more pronounced posterior pelvic tilt observed in SIF likely reduced anterior acetabular coverage and increased mechanical loading on the femoral head. This aligns with previous reports describing posterior pelvic tilt in cases of SIF and rapidly destructive coxarthrosis (Kubo et al., 2020). The frequent occurrence of SIF in individuals with bone fragility and without preceding trauma (Jo et al., 2016) suggests that both impaired subchondral bone quality and excessive mechanical stress contribute to its development.

In studies examining microdamage in the subchondral bone of the femoral head in femoral neck fractures, which are also fragility fractures occurring in elderly individuals, the values of BV/TV and Cr.Dn were reported to be comparable to those observed in the CNT group in the present study (Mori et al., 1997). In those reports, the mean age of elderly females without femoral neck fractures was 77 years, whereas those with femoral neck fractures was 78 years. Considering that the mean age of the CNT group in our study was 83 years, the Cr.Dn values observed in the CNT group appear to be reasonable, supporting the validity of our findings.

Histopathologically, SIF is characterized by irregular fracture callus, reactive cartilage, and granulation tissue (Yamamoto, 2012; Kawano et al., 2020). A previous case report has revealed bone loss and accumulation of microdamage in SIF (Iwata et al., 2019). Although microdamage also occurs in OA, the trabeculae in OA tend to be thick, whereas they are thin and sparse in SIF (Yamamoto, 2012; Yamamoto et al., 2000). In our study, both SIF and OA showed extensive microdamage with similar microdamage burden determined by Cr.S.Dn. However, compared to OA, the significantly longer Cr.Le values in SIF indicate greater crack propagation, suggesting Cr.Le may be a better indicator of potential fragility than Cr.Dn. Although longer cracks could alternatively reflect a more ductile response facilitating energy dissipation, the clinical presentation of SIF as a fragility fracture may favor the former interpretation. In this regard, it is interesting that Cr.Le is high in SIF, but BV/TV, although lower than in OA, is not different from the controls. This may suggest that the measurement of Cr.Le is a better measure of fragility than is BV/TV. That is, the fragility in SIF may have more to do with bone's material properties which may allow cracks to grow more easily. This observation is supported by fatigue loading studies demonstrating that microcracks can coalesce and propagate to form fracture lines (Vashishth et al., 2000; Vashishth et al., 1997; Iwata et al., 2014). These findings support the interpretation of SIF as a stress fracture arising from microdamage accumulation in fragile subchondral bone.

In contrast, OA secondary to acetabular dysplasia is associated with increased BV/TV due to bone modeling, allowing thickened trabeculae to accommodate multiple microcracks without significant propagation (Shimamura et al., 2019). Limited crack propagation in such cases may explain why subchondral collapse is less common in younger patients with SIF who have stronger bone structure and better material properties (bone quality) than older patients (Montero Furelos et al., 2024; Kim et al., 2016; Miyanishi et al., 2010). These data suggest the nature of the microdamage, not just the amount of the microdamage, is important to skeletal fragility. From a clinical perspective, the association between posterior pelvic tilt and microdamage accumulation observed in this study suggests that spinopelvic alignment assessment may help identify patients at high risk for SIF before collapse occurs. Patients with reduced lumbar lordosis and increased posterior pelvic tilt may warrant closer monitoring of bone quality and earlier intervention to prevent progression to rapidly destructive coxarthropathy.

This study had some limitations. First, this was a retrospective study, and the detailed patient background information for the cadaveric controls was unknown. Despite the possibility of bias resulting from unmeasured factors, there were no appreciable differences in the background characteristics of the patients in terms of sex or age. Second, because the femoral head of the SIF was not sampled immediately after disease onset, it was not possible to measure the accumulation of microdamage prior to crushing the femoral head. However, because only patients who have undergone surgery can have their femoral heads sampled, it is impractical to sample the femoral head before it collapses. Third, the cadaveric specimens were embalmed, which may have altered tissue properties compared with fresh specimens. However, as cadaveric donors were included only in histomorphometric analyses and not in radiological assessments, the potential influence of embalming on bone microstructure should be considered when interpreting the histological findings. Fourth, information on medications that may influence bone metabolism, such as NSAIDs, was not systematically recorded; three patients had received NSAIDs prior to surgery, which may have influenced the bone microstructure findings.

In conclusion, patients with SIF exhibited greater posterior pelvic tilt during the transition from supine to standing position and had a reduced lumbar lordosis compared to patients with OA. Histologically, SIF was associated with lower trabecular bone volume and longer microcracks. These data suggest the nature of the microdamage, not just the amount of the microdamage, is important to skeletal fragility. Our findings underscore the importance of assessing both spinopelvic alignment and bone quality when evaluating patients at risk for SIF. Future research should aim to develop early screening strategies for high-risk individuals and to investigate preventive interventions.

## CRediT authorship contribution statement

**Takahiro Negayama:** Writing – original draft, Data curation. **Ken Iwata:** Writing – review & editing, Conceptualization. **Masashi Shimamura:** Data curation. **Teppei Senda:** Data curation. **Ryuichi Isozaki:** Data curation. **Takanori Miki:** Supervision. **Masakazu Ishikawa:** Supervision.

## Declaration of Generative AI and AI-assisted technologies in the writing process

During the preparation of this work, the authors used Claude for language editing and grammar checking. After using this tool, the authors reviewed and edited the content as needed and take full responsibility for the content of the published article.

## Funding

This research did not receive any specific grant from funding agencies in the public, commercial, or not-for-profit sectors.

## Declaration of competing interest

The authors declare that they have no known competing financial interests or personal relationships that could have appeared to influence the work reported in this paper.

## Data Availability

Data will be made available on request.

## References

[bb0005] Bangil M., Soubrier M., Dubost J.J., Rami S., Carcanagues Y., Ristori J.M., Bussiere J.L. (1996). Subchondral insufficiency fracture of the femoral head. Rev. Rhum. Engl. Ed..

[bb0010] Burr D.B., Stafford T. (1990). Validity of the bulk-staining technique to separate artifactual from in vivo bone microdamage. Clin. Orthop. Relat. Res..

[bb0015] Chen M., Wang X., Takahashi E., Kaneuji A., Zhou Y., Kawahara N. (2022). Current research on subchondral insufficiency fracture of the femoral head. Clin. Orthop. Surg..

[bb0020] Chouhan V., Agrawal K., Vinothkumar T.K., Mathesul A. (2010). Bilateral insufficiency fracture of the femoral head and neck in a case of oncogenic osteomalacia. J. Bone Joint Surg. (Br.).

[bb0025] Dempster D.W., Compston J.E., Drezner M.K., Glorieux F.H., Kanis J.A., Malluche H., Meunier P.J., Ott S.M., Recker R.R., Parfitt A.M. (2013). Standardized nomenclature, symbols, and units for bone histomorphometry: a 2012 update of the report of the ASBMR Histomorphometry nomenclature committee. J. Bone Miner. Res..

[bb0030] Fazzalari N.L., Kuliwaba J.S., Forwood M.R. (2002). Cancellous bone microdamage in the proximal femur: influence of age and osteoarthritis on damage morphology and regional distribution. Bone.

[bb0035] Fazzalari N.L., Moore R.J., Manthey B.A., Vernon-Roberts B. (1989). Comparative study of iliac crest and proximal femur histomorphometry in normal patients. J. Clin. Pathol..

[bb0040] Fukui K., Kaneuji A., Fukushima M., Matsumoto T. (2014). Inversion of the acetabular labrum triggers rapidly destructive osteoarthritis of the hip: representative case report and proposed etiology. J. Arthroplast..

[bb0045] Hamada T., Yamamoto T., Shida J., Inokuchi A., Arizono T. (2014). Subchondral insufficiency fracture of the femoral head in a patient with alkaptonuria. Skeletal Radiol..

[bb0050] Higuchi Y., Tomosugi T., Futamura K., Okada M., Kusano T., Sawada H., Kobayashi K., Narumi S., Watarai Y., Goto N., Ando T., Sato K. (2022). Risk factors for subchondral insufficiency fracture of the femoral head in renal transplant patients. J. Bone Miner. Metab..

[bb0055] Ikemura S., Yamamoto T., Nakashima Y., Shuto T., Jingushi S., Iwamoto Y. (2005). Bilateral subchondral insufficiency fracture of the femoral head after renal transplantation: a case report. Arthritis Rheum..

[bb0060] Ishihara K., Miyanishi K., Ihara H., Jingushi S., Torisu T. (2010). Subchondral insufficiency fracture of the femoral head may be associated with hip dysplasia: a pilot study. Clin. Orthop. Relat. Res..

[bb0065] Iwasaki K., Yamamoto T., Nakashima Y., Mawatari T., Motomura G., Ikemura S., Iwamoto Y. (2009). Subchondral insufficiency fracture of the femoral head after liver transplantation. Skeletal Radiol..

[bb0070] Iwata K., Mashiba T., Hitora T., Yamagami Y., Yamamoto T. (2014). A large amount of microdamages in the cortical bone around fracture site in a patient of atypical femoral fracture after long-term bisphosphonate therapy. Bone.

[bb0075] Iwata K., Mashiba T., Shimamura M., Miki T., Yamamoto T. (2019). Accumulation of microdamage and low bone mass in the femoral head as a cause of subchondral insufficiency fracture in a patient with osteogenesis imperfecta. J. Bone Miner. Metab..

[bb0080] Jo W.L., Lee W.S., Chae D.S., Yang I.H., Lee K.M., Koo K.H. (2016). Decreased lumbar lordosis and deficient acetabular coverage are risk factors for subchondral insufficiency fracture. J. Korean Med. Sci..

[bb0085] Kanda Y. (2013). Investigation of the freely available easy-to-use software ‘EZR’ for medical statistics. Bone Marrow Transplant..

[bb0090] Kawano K., Motomura G., Ikemura S., Kubo Y., Hatanaka H., Utsunomiya T., Baba S., Nakashima Y. (2020). Subchondral insufficiency fracture of the femoral head in an elderly woman with symptomatic osteoarthritis of the contralateral hip. J. Orthop. Sci..

[bb0095] Kim S.M., Oh S.M., Cho C.H., Lim S.J., Moon Y.W., Choi S.H., Park Y.S. (2016). Fate of subchondral fatigue fractures of femoral head in young adults differs from general outcome of fracture healing. Injury.

[bb0100] Kubo Y., Motomura G., Ikemura S., Hatanaka H., Fukushi J.I., Hamai S., Yamamoto T., Nakashima Y. (2018). Osteoclast-related markers in the hip joint fluid with subchondral insufficiency fracture of the femoral head. J. Orthop. Res..

[bb0105] Kubo Y., Motomura G., Utsunomiya T., Fujii M., Ikemura S., Sonoda K., Nakashima Y. (2020). Distribution of femoral head subchondral fracture site relates to contact pressures, age, and acetabular structure. AJR Am. J. Roentgenol..

[bb0110] Miyanishi K., Ishihara K., Jingushi S., Torisu T. (2010). Risk factors leading to total hip arthroplasty in patients with subchondral insufficiency fractures of the femoral head. J. Orthop. Surg. (Hong Kong).

[bb0115] Montero Furelos L.A., De Castro Carrasco A., Cons Lamas S., Sanchez Sierra F.B., Caeiro-Rey J.R. (2024). Rapidly progressive osteoarthritis of the hip: a prospective study. J. Clin. Med..

[bb0120] Mori S., Harruff R., Ambrosius W., Burr D.B. (1997). Trabecular bone volume and microdamage accumulation in the femoral heads of women with and without femoral neck fractures. Bone.

[bb0125] Onishi E., Ota S., Fujita S., Tsukamoto Y., Yamashita S., Hashimura T., Matsunaga K., Yasuda T. (2022). Association between sagittal spinopelvic alignment and femoral head destruction in the early stage of rapidly destructive coxopathy. Bone Jt Open..

[bb0130] Shimamura M., Iwata K., Mashiba T., Miki T., Yamamoto T. (2019). Accumulation of microdamage in subchondral bone at the femoral head in patients with end-stage osteoarthritis of the hip. J. Bone Miner. Metab..

[bb0135] Sonohata M., Nakashima T., Kitajima M., Kawano S., Eto S., Mawatari M. (2021). Total hip arthroplasty using hydroxyapatite-coated cementless cup for rapidly destructive coxarthrosis: minimum 10-year follow-up. J. Orthop. Sci..

[bb0140] Taki N., Mitsugi N., Mochida Y., Akamatsu Y., Saito T. (2012). Change in pelvic tilt angle 2 to 4 years after total hip arthroplasty. J. Arthroplast..

[bb0145] Tamura S., Takao M., Sakai T., Nishii T., Sugano N. (2014). Spinal factors influencing change in pelvic sagittal inclination from supine position to standing position in patients before total hip arthroplasty. J. Arthroplast..

[bb0150] Uemura K., Takao M., Otake Y., Koyama K., Yokota F., Hamada H., Sakai T., Sato Y., Sugano N. (2017). Change in pelvic sagittal inclination from supine to standing position before hip arthroplasty. J. Arthroplast..

[bb0155] Vashishth D., Behiri J.C., Bonfield W. (1997). Crack growth resistance in cortical bone: concept of microcrack toughening. J. Biomech..

[bb0160] Vashishth D., Tanner K.E., Bonfield W. (2000). Contribution, development and morphology of microcracking in cortical bone during crack propagation. J. Biomech..

[bb0165] Yamamoto T. (2012). Subchondral insufficiency fractures of the femoral head. Clin. Orthop. Surg..

[bb0170] Yamamoto T., Bullough P.G. (1999). Subchondral insufficiency fracture of the femoral head: a differential diagnosis in acute onset of coxarthrosis in the elderly. Arthritis Rheum..

[bb0175] Yamamoto T., Yamaguchi T., Lee K.B., Bullough P.G. (2000). A clinicopathologic study of osteonecrosis in the osteoarthritic hip. Osteoarthr. Cartil..

